# Supplementary material for: Bayesian refinement of protein structures and ensembles against SAXS data using molecular dynamics
Source: PLoS Comput Biol. 2017 Oct 18;13(10):e1005800. doi: 10.1371/journal.pcbi.1005800 (PMC5662244; doi:10.1371/journal.pcbi.1005800)
Supplement: S1 Archive — (BZ2) [file pcbi.1005800.s011.tar.bz2 › gromacs_4.62_bayesian_saxs_refi/share/html/online/g_lie.html]

g\_lie


| g\_lie Main Table of Contents |

**VERSION 4.6  
Sat 19 Jan 2013**

---

### Description

g\_lie computes a free energy estimate based on an energy analysis
from. One needs an energy file with the following components:
Coul (A-B) LJ-SR (A-B) etc.

### Files

| option | filename | type | description |
| --- | --- | --- | --- |
| **-f** | ener.edr | Input | Energy file |
| **-o** | lie.xvg | Output | xvgr/xmgr file |

### Other options

| option | type | default | description |
| --- | --- | --- | --- |
| **-[no]h** | bool | no | Print help info and quit || **-[no]version** | bool | no | Print version info and quit || **-nice** | int | 19 | Set the nicelevel || **-b** | time | 0 | First frame (ps) to read from trajectory || **-e** | time | 0 | Last frame (ps) to read from trajectory || **-dt** | time | 0 | Only use frame when t MOD dt = first time (ps) || **-[no]w** | bool | no | View output .xvg, .xpm, .eps and .pdb files || **-xvg** | enum | xmgrace | xvg plot formatting: xmgrace, xmgr or none || **-Elj** | real | 0 | Lennard-Jones interaction between ligand and solvent || **-Eqq** | real | 0 | Coulomb interaction between ligand and solvent || **-Clj** | real | 0.181 | Factor in the LIE equation for Lennard-Jones component of energy || **-Cqq** | real | 0.5 | Factor in the LIE equation for Coulomb component of energy || **-ligand** | string | none | Name of the ligand in the energy file |

---

http://www.gromacs.org  
gromacs@gromacs.org
